# Repurposing glucocorticoids as adjuvant reagents for immune checkpoint inhibitors in solid cancers

**DOI:** 10.20892/j.issn.2095-3941.2021.0491

**Published:** 2021-10-22

**Authors:** Yingyan Yu

**Affiliations:** 1 Department of General Surgery, Ruijin Hospital, Shanghai Institute of Digestive Surgery, Shanghai Key Laboratory for Gastric Neoplasms, Shanghai Jiao Tong University School of Medicine, Shanghai 200025, China

Glucocorticoids are drugs that are controversial in the treatment of malignant tumors, especially in regimens using immune checkpoint inhibitors. Some oncologists warn that glucocorticoids might affect or even weaken the therapeutic efficacy of PD-1 inhibitors. Recently, Xiang et al.^1^ identified drugs that could target the dual-target of PD-L1/IDO1, and published their finding of “Dexamethasone suppresses immune evasion by inducing GR/STAT3 mediated downregulation of PD-L1 and IDO1 pathways” in *Oncogene*, a leading international oncology journal. Clinicians, especially oncologists, are interested in knowing what types of cancers are indicators of the drug and what dosage of dexamethasone might be appropriate for assisting cancer immune therapy. Here, the author provides a review of previous reports and their potential value for suggesting treatments involving immune checkpoints inhibitors.

## Drug repurposing, a new direction for pharmaceutical research

It is well-known that development of new drugs from the beginning is a time-consuming and laborious process, and it therefore cannot meet all urgent needs. Since the strategy of drug repurposing was proposed, it has been favored by research communities^2^. Drug repurposing (also known as drug repositioning) means “old drugs, new uses”, which refers to using drugs that have been approved by the U.S. Food and Drug Administration (FDA) for clinical use or in clinical trials with potential use on new targets. It provides old drugs with new uses^3,4^. The advantages of this strategy are speed, safety, and low cost, because drugs and chemicals have passed the tests of drug toxicology and pharmacokinetics. These drugs are less risky than developing new drugs, which may have toxic side effects. Drug repurposing research highly relies on bioinformatics and chemical informatics^5^. Recently, Li et al.^6^ characterized the mechanisms of acquired resistance to gefitinib in non-small cell lung cancer. They analyzed microarray data of an acquired gefitinib-resistant cell line (PC9GR) and a gefitinib-sensitive cell line (PC9), and constructed a differentially expressed gene set. Using CMap database screening, small molecule drugs with the potential to overcome drug resistance, such as emetine and cephaeline, were found to increase the sensitivity to gefitinib. The molecular mechanism may be related to the regulation of PI3 and S100A8^6^. Xiang et al.^1^ also adopted this strategy; they identified a group of compounds that may simultaneously inhibit PD-L1 and IDO1, and among them, dexamethasone was the optimal drug. The strategy of drug repurposing has also played an important role in the COVID-19 pandemic^7^. Because there are no treatment options available for this deadly contagious disease, scientists rapidly screened for effective drugs. For example, Han et al.^8^ identified 200 drugs predicted to target SARS-CoV-2-induced signaling pathways, and 40 of them are already in COVID-19 clinical trials, testifying to the validity of this approach. They found that the drugs, proguanil and sulfasalazine, could inhibit viral replication in cell assays.

Along with the rapid development of human genomics, a large amount of genomic data has been submitted to public databases, which provide researchers with valuable resources for drug screening. Cancer Cell Line Encyclopedia (CCLE), The Cancer Genome Atlas (TCGA), and Connectivity Map (CMap) are important open databases. CCLE is a collection of transcriptome information before and after treating 1,019 cancer cell lines originating from 26 types of cancers by 24 kinds of compounds^9^. TCGA consists of multi-omics data of more than 11,000 cases from 33 types of cancers^10^. CMap is a database of transcriptome information before and after treating 9 human cancer cell lines with 2,837 compounds^11^. Xiang et al.^7^ reported that cross-database analysis was an important procedure. They focused on multiple immune-related molecules, and a total of 25 potential compounds that may reverse tumor immune evasion were identified. Among them, dexamethasone showed an anti-tumor effect on the cancer cell lines-T cells co-cultured system, the three-dimensional organoid-T cells co-cultured system, and the humanized immune microenvironment mouse model. Dexamethasone-mediated transcriptional suppression of PD-L1 and IDO1 depended on the nuclear translocation of the GR/STAT3 complex. Their study suggested that dexamethasone could be used as a sensitization reagent for immune checkpoint inhibitors^1^.

## High expression of PD-Ll/IDO1 in several types of solid cancers

Theoretically, combination therapy for dual targets should increase the therapeutic effect, but the immune-related adverse events caused by multi-type immune checkpoint inhibitors should not be ignored^12^. To determine whether solid cancers are suitable for combination therapy of dual-targets, Xiang et al.^1^ conducted correlation analyses of multiple immune evasion-related genes including *BTLA*, *VISTA*, *CD160*, *PD-L1*, *CTLA4*, *IDO1*, *LAG3*, *LGALS9*, *TNFRSF14*, and *VTCN1*, and *PD-L1* and *IDO1* showed the strongest correlations. High coefficients were found in gastric cancers and colorectal carcinomas. In addition, other solid cancers such as skin cancers, lung cancers, pancreatic cancers, and breast cancers also showed high expressions of PD-Ll/IDO1. Using transcriptomics data analysis between PD-L1(−)/IDO1(−) and PD-L1(+)/IDO1(+), a differentially-expressed gene set was used for CMap screening. Twenty-five compounds were predicted as potential inhibitors for PD-L1 and IDO1 dual-targets (**Table 1**). Although dexamethasone and TWS-119 showed inhibitory effects on PD-L1 and IDO1 in a dose-dependent manner on gastric cancer cell lines, the efficacies of other chemicals on different types of cancers are worthy of further validation.

**Table 1:** The 25 predicted candidates targeted to the PD-L1/IDO1 dual-target system

Chemicals	Original functions
Androstenol	A postulated pheromone in male
Tipifamib	Farnesyl transferase inhibitor
Verapamil	Calcium channel blocker
IB-MECA	Selective A3 adenosine receptor (A3AR) agonist
WH-4023	Selective dual LCK/SRC inhibitor
Atorvastatin	HMG CoA reductase inhibitors, Statin-class
PD-0325901	Selective and non-ATP-competitive MEK inhibitor
EMD-386088	5-HT6 receptor agonist
TWS-119	GSK-3β inhibitor
Fostamatinib	Spleen tyrosine kinase (SYK) inhibitor
CC-930	JNK inhibitor
Lapatinib	EGFR, ERBB2 inhibitor
SB-216763	GSK-3 inhibitor
RITA	p53-HDM-2 interaction inhibitor
PD-0325901	MEK inhibitor
PKC beta-inhibitor	Blocking PKC beta
RHO-kinase-inhibitor-iii	Rho-kinase inhibitor and vasodilator
Triacsin-c	Production of Streptomyces aureofaciens, ACSL inhibitor
TTNPB	RAR agonist
AS-703026	MEK1/2 inhibitor
Eicosatetranoic-acid	Cyclooxygenase and lipoxygenases inhibitor
PD-98059	MEK inhibitor
Dexamethasone	Glucocorticoid
PP-1	SRC inhibitor
AC-55649	RARβ2 receptor agonist

## Glucocorticoid-related gene regulation

Glucocorticoids (i.e., cortisone) play roles in combination with their glucocorticoid receptors (GRs), which are encoded by the *NR3C1* gene. Upon glucocorticoid binding, GR undergoes conformational changes, and translocates into the nucleus to bind to the promoters of target genes^13^. Ayroldi et al.^14^ proposed that among the GR-induced genes, glucocorticoid-induced leucine zipper (GILZ) mediated various anti-inflammatory effects. GILZ affects the immune system, tumor microenvironment, and cancer cell biology. Kumar et al.^15^ found that treatment with dexamethasone significantly increased miR-708 expression by transactivation of GR in MCF-7 and MDA-MB-231 breast cancer cell lines. GR agonist treatment or miR-708 mimic transfection remarkably suppressed the activity of nuclear factor-kappa B and its downstream targets, including COX-2, c-MYC, CCND1, MMP-2, MMP-9, CD24 and CD44, which are known to be involved in cell proliferation, cell-cycle progression, and metastasis. Their study suggested that GR agonists induced miR-708 and downstream suppression of NF-kappa B signaling, which may be applicable as a novel therapeutic intervention in breast cancer treatment^15^. Shen et al.^16^ integrated multi-omics data to identify the responsive genes of dexamethasone and found that they were transcriptionally down-regulated. In the cancer microenvironment, the response score of dexamethasone is positively correlated with a subset of innate immune cells. This study indicates that dexamethasone is potentially correlated with anti-cancer immunity in the cancer microenvironment^16^.

Gene polymorphism is a factor that affects the sensitivity of glucocorticoid treatments. Peng et al.^17^ found that G1199A polymorphism of the *ABCB1* gene altered the sensitivity of steroids in LLC-PK1 pig kidney cells. In the treatment of bronchopulmonary dysplasia of infants, the clinical efficacy of corticosteroid on the severity of lung disease is highly variable. Using multivariate linear regression analysis, Lewis et al.^18^ conducted a pharmacogenetic analysis of data from a large randomized controlled trial in infants treated with dexamethasone or hydrocortisone. They found that rs7225082 in the intron of the *CRHR1* gene was significantly associated with the magnitude of the decrease. The T allele at rs7225082 is associated with less response to corticosteroids, indicating that genetic variability is associated with the responsiveness to corticosteroid. Identification of genetic markers of the responsiveness of corticosteroid may help with individual therapies, and may optimize the risk-to-benefit ratio in an individual therapy^18^.

## Glucocorticoids reshape the tumor microenvironment

Targeting the tumor microenvironment is a new direction in solid cancer therapy. Glucocorticoids functions as anti-inflammatory agents in treating many diseases, including cancers, and it helps manage various side effects of chemotherapy, radiotherapy, and immunotherapy. Recently, Geng et al.^19^ constructed a nanosystem for increasing drug penetration in solid cancers. The nanosystem consisted of tannic acid, carrier ZIF-8, encapsulated glucose oxidase, and dexamethasone. The loaded dexamethasone expanded the pores of the nucleus to promote glucose oxidase entering the nucleus, solving the shortcomings of the short life of reactive oxygen species, but it also inhibited the production of collagen, to reshape the tumor microenvironment and inhibit cancer cell metastasis. Because dexamethasone can enlarge the nuclear pores and promote the entry of drugs into the nucleus, using dexamethasone could be useful in clinical applications^19^. Martin et al.^20^ studied the effect of dexamethasone on normalizing the tumor microenvironment in a metastatic murine breast cancer model. Dexamethasone normalized vessels and the extracellular matrix, to reduce interstitial fluid pressure, tissue stiffness, and solid stress. In turn, the penetration of 13 and 32 nm nanocarriers was increased by changing interstitial hydraulic conductivity, resulting in accumulation of 30 nm polymeric micelles containing cisplatin, to treat murine models. Together, these results suggested that pretreatment with dexamethasone before nanocarrier administration could increase the efficacy against cancers^20^.

## Dosage concerns of glucocorticoids in cancer therapy

Glucocorticoids are used as anticancer drugs for lymphohematopoietic malignancies, and for controlling the side effects of chemo/radiotherapy in solid tumors. Glucocorticoids can increase appetite, reduce weight loss, alleviate fatigue, relieve pain, and prevent vomiting^21,22^. Some studies suggest that the use of glucocorticoids in advanced cancers should be avoided, because the drug may reduce survival of patients with lung cancers^23^. It is clear that using glucocorticoids for the treatment of cancers has both positive and negative effects^24^. The molecular mechanisms underlying the effects of glucocorticoids are numerous, but not all of them have been determined. Xiang et al.^1^ reported that responses to glucocorticoid differ, based on the cancer type. The response to glucocorticoid is also associated with the expression level of GR, gene polymorphism, and the tumor immune microenvironment. Using preclinical models, Yao et al.^25^ showed that dexamethasone inhibited pancreatic tumor growth. Dexamethasone treatment significantly inhibited colony formation, migration, and tumor growth of PANC-1 cells. The underlying mechanisms involved suppression of phosphorylation of nuclear factor κB and downregulation of the epithelial-to-mesenchymal transition, interleukin 6, and vascular endothelial growth factor^25^. Pang et al.^26^ evaluated the potential of dexamethasone in the treatment of breast cancer. They found that the administration of low dose dexamethasone (5 µg/kg) suppressed tumor growth and distant metastasis in the MCF-7 and MDA-MB-231 xenograft mouse model, whereas treatment with high dose dexamethasone (100 µg/kg) enhanced tumor growth and metastasis. The dexamethasone-mediated inhibition of cell adhesion, migration, and invasion was partly through induction of microRNA-708 and subsequent RAP1B-mediated signaling in MDA-MB-231 cells. The results revealed that dexamethasone acted as a double-edged sword in breast cancer progression and metastasis^26^. Lin et al.^27^ used low dose dexamethasone (5 µg/kg) to suppress tumor growth and distant metastasis in a mouse model of ovarian cancer. Xiang et al.^1^ used a NOD/SCID mouse model with a humanized immune microenvironment. The cancer inhibitory efficacy of low dose dexamethasone (0.1 mg/kg) combined with pembrolizumab administration (5 mg/kg) was observed in both gastric cancer and pancreatic cancer models^1^. Although administration of low dose glucocorticoid has already been described in this review, in cancer therapy, their concentrations varied, probably due to the differences in the types of cancers, different animal models, and even different batches of drugs. Because these studies were conducted in laboratories, more studies will be needed to elucidate the optimal dose, timing, and administrative routes, using clinical studies to develop a patient-specific strategy for administering glucocorticoids.

## Conclusions

The therapeutic efficacy of immune checkpoint inhibitors has prompted increasing studies by the cancer research community^28^. Therapeutic resistance in some patients has prompted researchers to characterize underlying molecular mechanisms and identify alternative new drugs. The strategy of targeting multiple immune checkpoints has been extensively studied, but a further understanding of the molecular mechanisms is needed. The drug repurposing approach accelerates the pace of cancer immune therapy. Glucocorticoids are widely used as supportive treatments to reduce the side effects of chemotherapy. Their anti-cancer functions, especially their efficacy for immune checkpoint inhibitors in solid cancers, warrant further studies.
